# Alternative NF-κB Isoforms in the *Drosophila* Neuromuscular Junction and Brain

**DOI:** 10.1371/journal.pone.0132793

**Published:** 2015-07-13

**Authors:** Bo Zhou, Scott A. Lindsay, Steven A. Wasserman

**Affiliations:** Section of Cell & Developmental Biology, Division of Biological Sciences, University of California San Diego, La Jolla, California, United States of America; Duke University Medical Center, UNITED STATES

## Abstract

The *Drosophila* NF-κB protein Dorsal is expressed at the larval neuromuscular junction, where its expression appears unrelated to known Dorsal functions in embryonic patterning and innate immunity. Using confocal microscopy with domain-specific antisera, we demonstrate that larval muscle expresses only the B isoform of Dorsal, which arises by intron retention. We find that Dorsal B interacts with and stabilizes Cactus at the neuromuscular junction, but exhibits Cactus independent localization and an absence of detectable nuclear translocation. We further find that the Dorsal-related immune factor Dif encodes a B isoform, reflecting a conservation of B domains across a range of insect NF-κB proteins. Carrying out mutagenesis of the *Dif* locus via a site-specific recombineering approach, we demonstrate that Dif B is the major, if not sole, Dif isoform in the mushroom bodies of the larval brain. The Dorsal and Dif B isoforms thus share a specific association with nervous system tissues as well as an alternative protein structure.

## Introduction

Signal-regulated nuclear import of NF-κB proteins regulates gene expression in a wide range of invertebrates and vertebrates, including humans. Among the best-studied examples are mammalian NF-κB and *Drosophila* Dorsal [1–3]. Prior to signaling, an inhibitory IκB protein binds to the NF-κB protein, retaining it in the cytoplasm. Activation of an upstream signaling pathway triggers inhibitor phosphorylation and degradation, freeing the NF-κB protein for translocation into the nucleus. There, the NF-κB protein functions as a transcription factor: the amino-terminal Rel Homology Region (RHR) mediates homodimerization and DNA binding, whereas the carboxyl-terminal activation domain directs target gene expression.

In *Drosophila*, the NF-κB family members Dorsal and Dif, the Dorsal-related immune factor, act in several physiological contexts. Early in development, a nuclear concentration gradient of Dorsal across the syncytial embryo establishes the dorsoventral body axis [4–6]. In postembryonic stages, Dorsal and Dif in the fat body activate innate immune genes in response to a range of microbial infections [7–10]. In both settings, these transcription factors are regulated by the transmembrane receptor Toll and by the IκB-related inhibitor Cactus (for review, see [11]). Dorsal and Dif are also expressed in non-immune tissues of the larva. Dorsal is expressed in the larval neuromuscular junction (NMJ) and, more specifically, at the subsynaptic reticulum (SSR), a highly convoluted muscle membrane structure that surrounds synaptic boutons [12–15]. Dif, in contrast, is found in the larval brain [13].

Alternative splicing of the *dorsal* (*dl*) gene gives rise to a pair of protein isoforms that differ in their carboxyl-terminal domains [16]. The A isoform is expressed maternally and zygotically, is responsible for dorsoventral axis formation, and, with Dif, mediates the Toll-regulated innate immune response [10, 17, 18]. In contrast, the B isoform is only expressed zygotically and has no defined involvement in development or immunity [16]. Here we describe tissue-specific alternative splicing and noncanonical localization and regulation of the B isoform of Dorsal and, as well, a B isoform of Dif.

## Results

### The larval NMJ expresses exclusively an alternative Dorsal isoform

**Fig 1:**
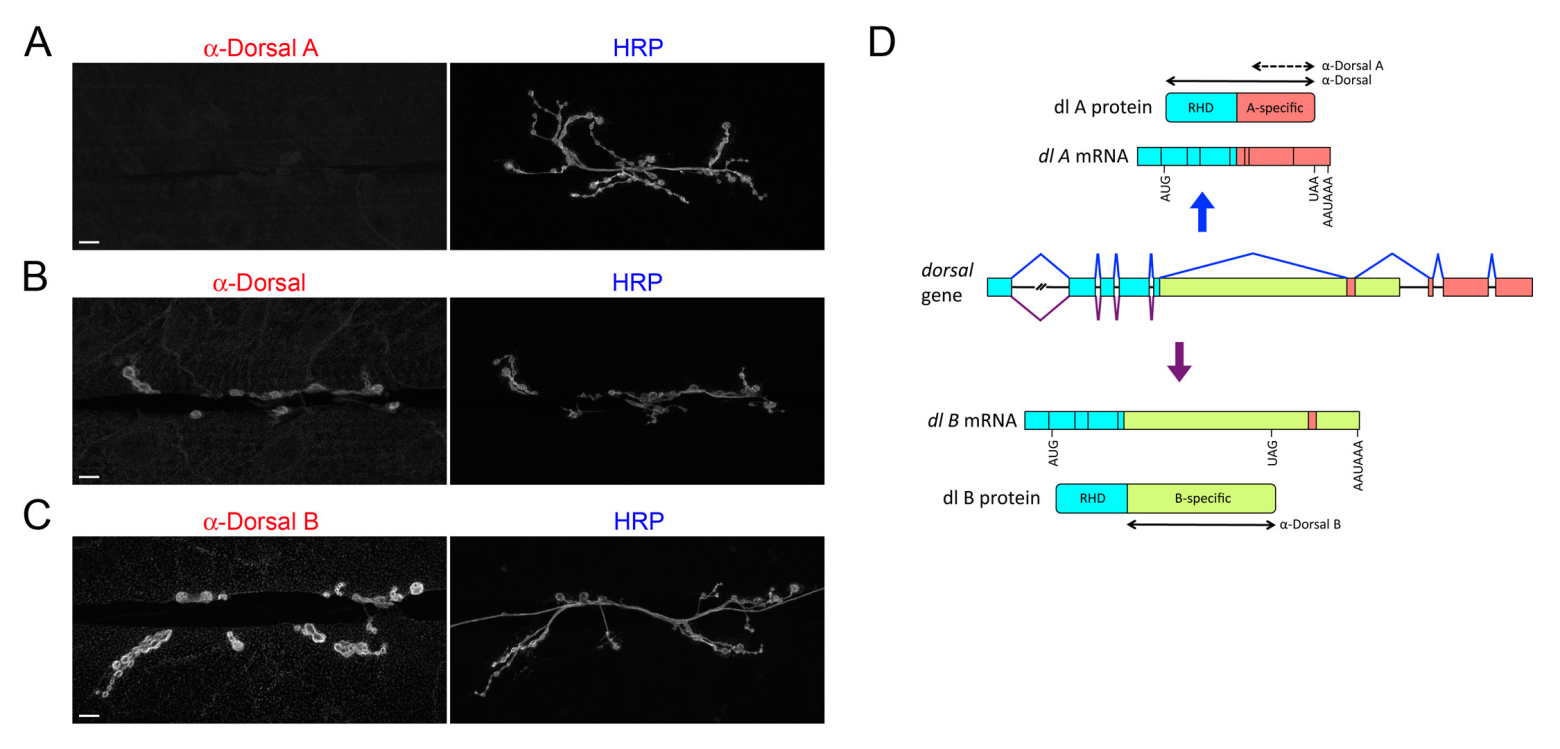
Dorsal B is the sole *dorsal* isoform expressed at the larval NMJ. NMJs from wild-type (*w* ^*1118*^) larvae were labeled with (A) α-DorsalA, (B) α-Dorsal, or (C) α-DorsalB. All samples were also stained with α-HRP, which recognizes a carbohydrate epitope on neuronal membranes, including axons at the NMJ [21, 22]. Images are of muscle 6/7. Scale bar = 10 μm. (D) Schematic of alternative splicing of *dorsal* mRNA and resulting A and B protein isoforms. Double-headed solid arrows indicate the protein regions used as antigens to generate α-Dorsal and α-DorsalB antibodies. The double-headed dotted arrow indicates the region to which the epitope recognized by α-DorsalA has been mapped [19].

Our discovery of tissue-specific Dorsal isoform expression grew out of our immunohistochemical studies of the NMJ. In particular, we noted that a monoclonal antibody that recognizes an epitope specific to Dorsal A [19] gave no signal at the NMJ (Fig 1A), although it readily detected Dorsal A in a transgenic larval tissue (S1 Fig). In contrast, our polyclonal α-Dorsal serum [20] for which the antigen included the Rel homology domain (RHD) common to the A and B isoforms (Fig 1D) gave a strong signal at the NMJ (Fig 1B). Taken together, these results led us to hypothesize that the *Drosophila* NMJ expresses Dorsal B but not Dorsal A.

*To test the hypothesis that only the B isoform of* dorsal *is expressed at the larval NMJ, we generated antibodies against the B-specific domain* (*see* Fig 1D; S1 File). *Staining with these* α-*DorsalB antibodies resulted in a strong signal at the larval NMJ* (Fig 1C). *Furthermore, the staining pattern was indistinguishable from that observed with* α*-Dorsal (compare* Fig 1B and 1C). *We conclude that the Dorsal expression previously detected at the NMJ* [12, 15] *is in fact exclusively due to the B isoform. In addition, we note that the NMJ is the first* Drosophila *tissue shown to express Dorsal B and not Dorsal A*.

### The presence of Cactus at the NMJ is dependent on Dorsal B

The inhibitory IκB protein Cactus is found at all known sites of Dorsal expression, including the NMJ [12, 23]. In general, IκB proteins are stable when bound to an NF-κB protein, but rapidly degraded in the absence of such interaction (see, for example, [24]). Thus, in early embryos, which express only Dorsal A, a mutation that eliminates Dorsal expression triggers a concomitant loss of Cactus [25, 26].

**Fig 2:**
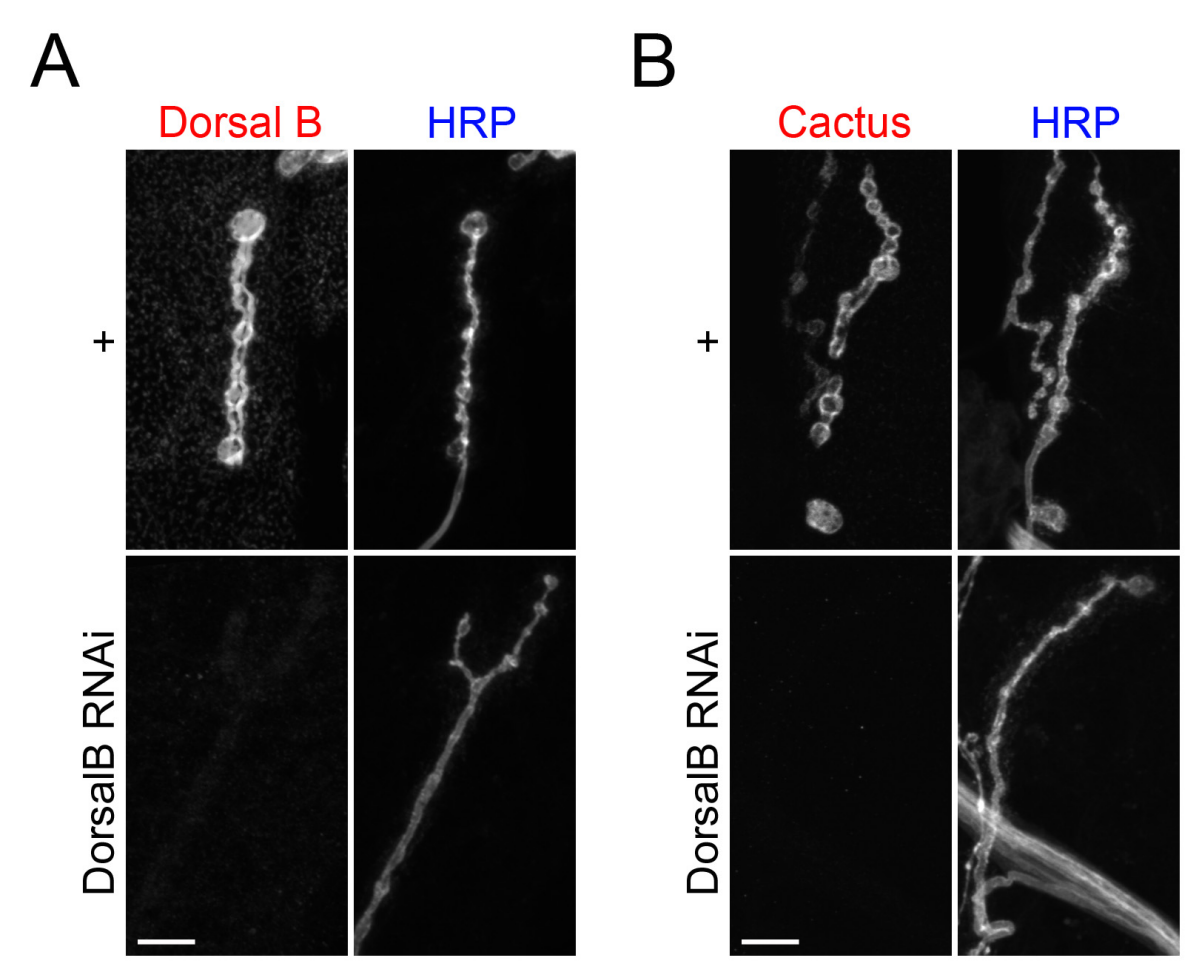
Dorsal B stabilizes Cactus at the larval NMJ. NMJs from + (*24BGal4/+*) and DorsalB knockdown (*24BGal4/*UAS-DorsalB-RNAi) larvae labeled with (A) α-DorsalB or (B) α-Cactus, as well as α-HRP. Images are of muscle 4 and scale bar = 10 μm.

The ankyrin repeats of Cactus bind to the RHR domain that is found in both Dorsal A and B [19, 25, 27, 28]. We therefore expected that Dorsal B, like Dorsal A, would bind to and stabilize Cactus. To test this prediction, we used a Gal4 driver (24B) and a UAS-directed short hairpin RNA to knock down Dorsal B expression specifically in larval muscle cells. As shown in Fig 2A, this use of RNA interference (RNAi) was highly effective, reducing Dorsal B expression in larval muscle below the limit of detection.

Taking advantage of the efficient RNAi against Dorsal B, we examined Cactus expression in the presence and absence of Dorsal B (Fig 2B). Using an α-Cactus serum, we readily detected Cactus in wild-type muscle preparations, where it exhibited the same localization pattern as Dorsal B. When Dorsal B expression was knocked down, however, the Cactus signal was absent. We conclude that interaction with Dorsal B is required for stable expression of Cactus protein in larval muscle.

### Dorsal B remains localized outside of nuclei in the absence of Cactus

**Fig 3:**
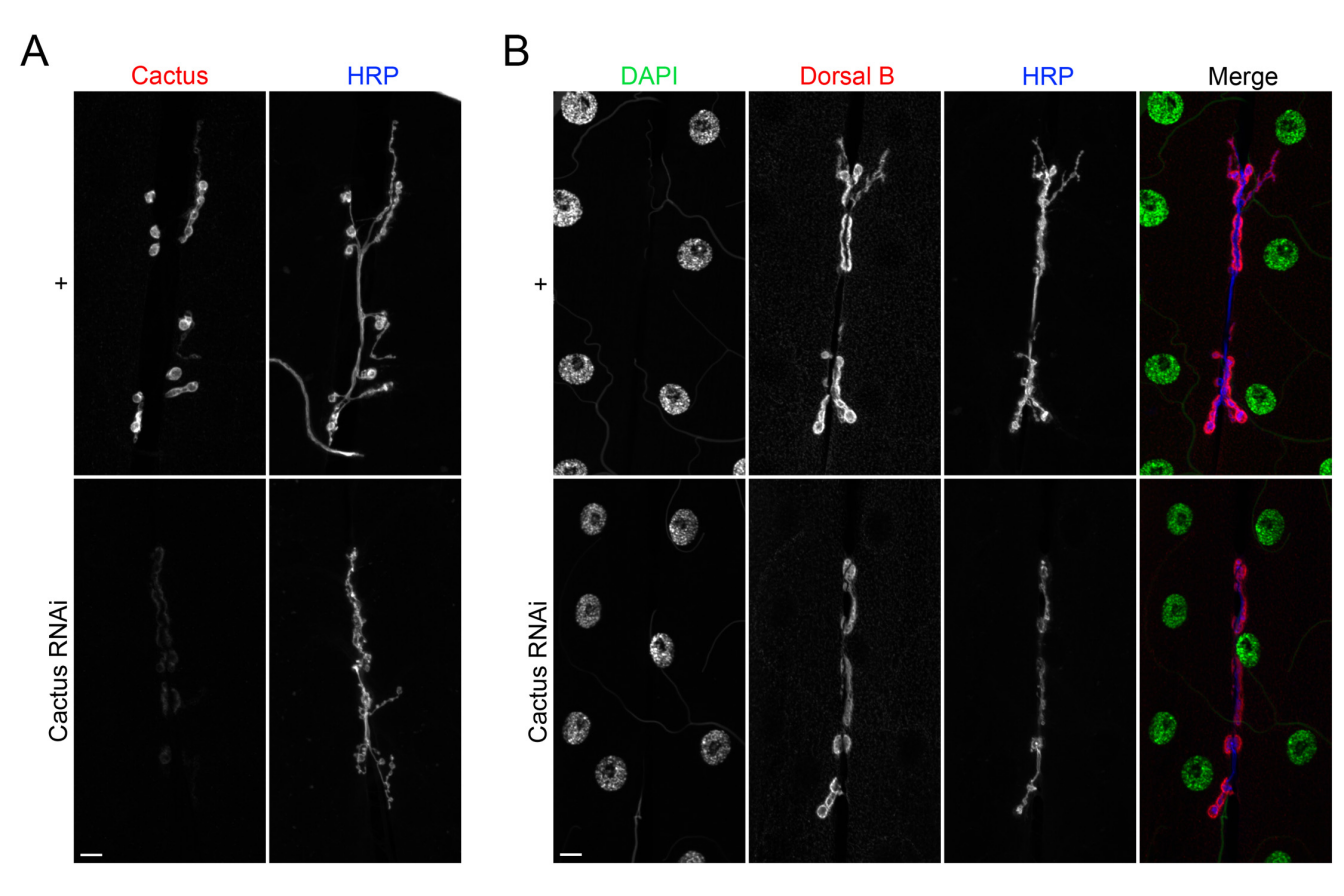
Removing Cactus does not trigger nuclear translocation of Dorsal B. NMJs from + (*24BGal4/+*) and Cactus RNAi (*24BGal4/*UAS-Cactus-RNAi) larvae labeled with (A) α-Cactus or (B) α-DorsalB and DAPI, as well as with α-HRP. Images are of muscle 6/7. Scale bar = 10 μm.

Cactus localizes with and interacts with Dorsal B at the NMJ. Does Cactus regulate nuclear translocation of Dorsal B, as it does for Dorsal A in other tissues? To answer this question, we carried out colocalization studies, using DAPI staining to delineate nuclei. As shown in Fig 3, there was no detectable nuclear localization of Dorsal B in the wild-type NMJ. When we knocked down Cactus expression, RNAi was efficient but Dorsal B localization was unchanged. The absence of Dorsal B nuclear localization in larval muscle upon Cactus elimination is in marked contrast to the behavior of Dorsal A in embryos and the larval fat body.

We draw two conclusions from the experiment shown in Fig 3. First, although both isoforms of Dorsal bind to Cactus, only the A isoform translocates into nuclei when Cactus is absent. Second, Cactus is not required for the specific, extranuclear localization of Dorsal B at the NMJ.

### Dorsal B colocalizes with Discs large (Dlg) at the SSR

**Fig 4:**
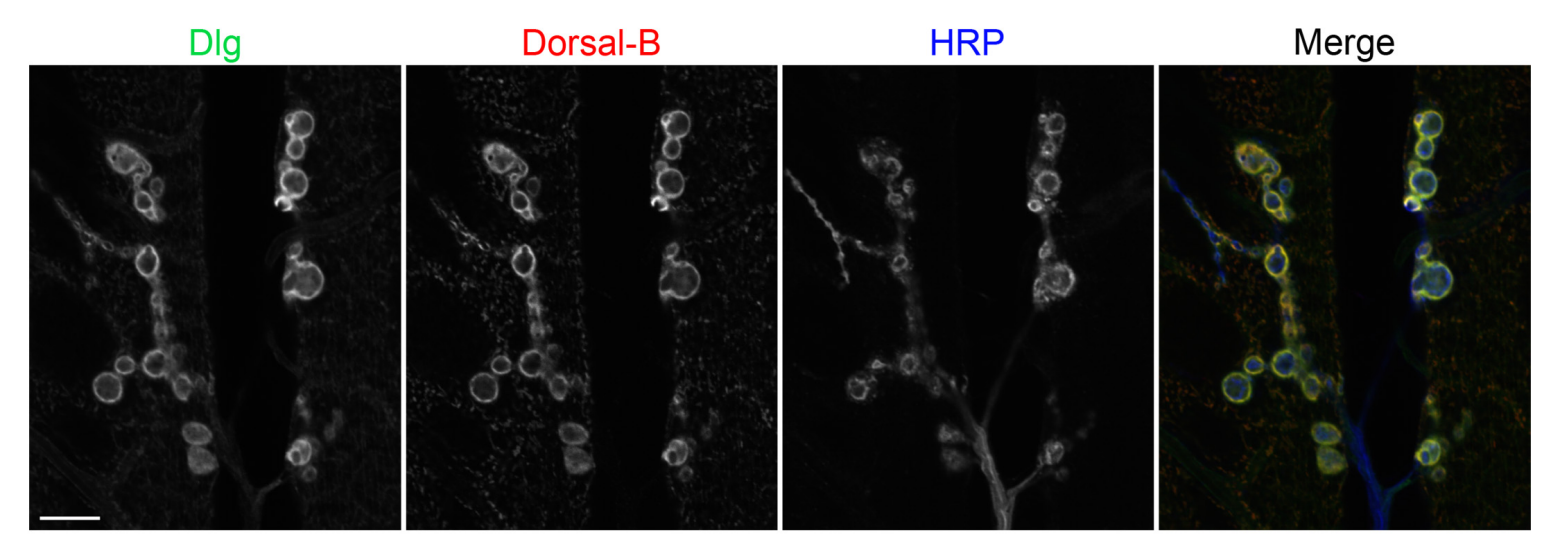
Dorsal B colocalizes with Dlg at the SSR. Body wall muscles from *w* ^*1118*^ larvae were labeled with antibodies to Discs large protein (α-Dlg), α-DorsalB, and α-HRP. Images are of muscle 6/7 and scale bar = 10 μm.

To further resolve the pattern of Dorsal B expression, we carried out colocalization studies with antibodies directed at Dorsal B and at Discs large (Dlg), a membrane associated guanylate kinase that contributes to SSR organization [29–31]. As shown in Fig 4, Dorsal B and the Dlg protein colocalized at the SSR. However, when we blocked Dlg expression at the SSR, we found no change in Dorsal B localization, indicating that the known scaffolding role of Dlg does not include a required role in recruitment or retention of Dorsal B (S2 Fig).

### The mushroom bodies of the brain express a B isoform of Dif

In the course of studying alternative splicing of *dorsal*, we discovered that the neighboring and closely related *Dif* gene also undergoes alternative splicing to produce a B isoform (S3 Fig), as apparent in RNAseq data from the modENCODE project (flybase.org). Comparison of predicted amino acid sequences for the *D*. *melanogaster* Dif and Dorsal isoforms revealed that the B specific domain is 40% identical across 530 residues (S4, S5, and S6 Figs). This degree of conservation is close to that of the RHD (48% identity) and much higher than that observed for the A domain (less than 20% overall identity).

**Fig 5:**
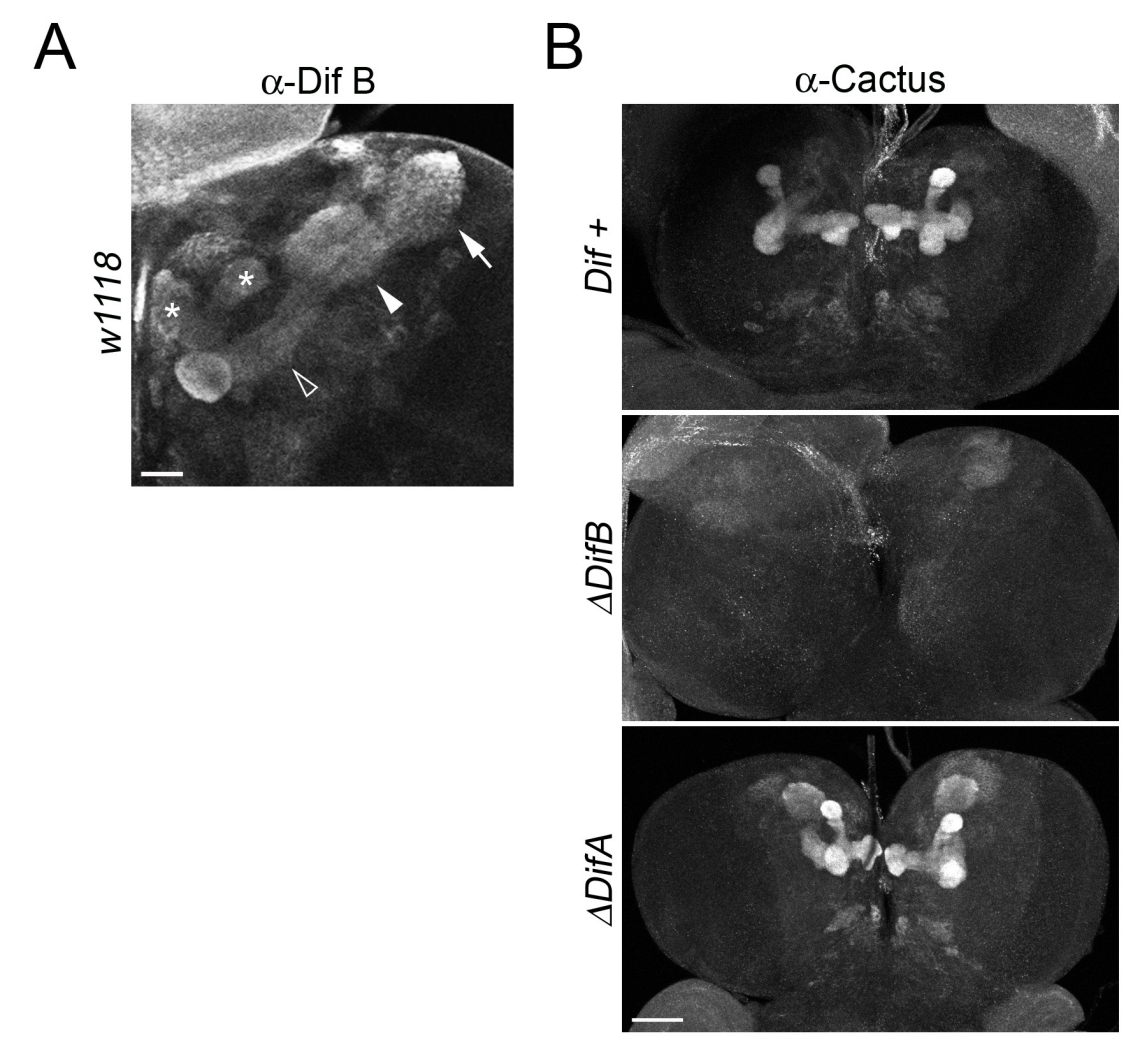
Dif B expressed in larval mushroom bodies stabilizes Cactus. (A) The brain from a wild-type (*w* ^*1118*^) larva stained with α-DifB. Asterisks, vertical and medial lobes; hollow arrowheads, pedunculus; filled arrowheads, calyx; arrows, cell body. Scale bar = 20 μm. (B) Cactus is absent from the mushroom bodies when expression of *Dif B*, but not *Dif A*, is blocked. Brains from *Dif +* (*DfJ4/DfExel7068; J4rescue*), *ΔDifA* (*DfJ4/DfExel7068; J4rescue-ΔDifA*), and *ΔDifB* (*DfJ4/Exel7068; J4rescue-ΔDifB*) larvae labeled with α-Cactus. Scale bar = 50 μm.

Dif, like Dorsal, has been found to be expressed in tissues associated with the nervous system. Specifically, Dif has been found in the larval brain, colocalizing with Cactus in a pair of structures, the mushroom bodies, that have an important role in associative learning and memory [13]. To examine whether this expression might be due to Dif B, we generated a domain-specific, α-DifB serum. Staining of larval brains with these antibodies revealed that Dif B is in fact expressed in the paired mushroom bodies of the larval brain. We detected Dif B throughout the entire mushroom body, i.e., in the cell body, calyx, pedunculus, and both vertical and medial lobes (Fig 5A).

### Dif B stabilizes Cactus in mushroom bodies

Does Dif B interact with and stabilize Cactus in the brain? To address this question, we used recombineering in combination with germline transformation to complement deficiencies spanning the *Dif* gene with a wild-type transgene (*J4rescue*) or a transgene specifically blocked for production of the A or B splice form *(ΔDifA* or *ΔDifB*, S7 Fig). We then stained the mutant lines with our α-Cactus serum and found that Cactus expression was wild type in the mushroom bodies of both *J4rescue* and *ΔDifA* animals, but absent from the mushroom bodies of *ΔDifB* larvae (Fig 5B). We conclude that Dif B in the larval brain binds to and stabilizes Cactus and, furthermore, that Dif B is the major, if not sole, form of Dif in the larval mushroom bodies.

## Discussion

### Conservation of B isoforms of invertebrate NF-κB proteins

Coordinated changes in mRNA processing give rise to the B isoforms of both the *dorsal* and *Dif* genes. Specifically, alternative splicing in the form of intron retention is coupled with utilization of alternative transcription termination and polyadenylation sites (see S3 Fig). Not only do both genes exhibit intron retention, but they also have a common exon and intron organization, share similarity in encoded protein sequence, have overlapping functions in innate immunity, and are found in close proximity in the genome. In light of these observations, we envision that Dorsal and Dif arose by duplication of a common ancestor that also encoded an A and B isoform. This duplication appears to be quite recent, since we have not found Dif orthologs outside of the *Drosophila* genus.

Intron retention and an alternative transcription termination site have also been described for *cactus* [32]. As with *dorsal* and *Dif*, *cactus* expresses the alternative isoform zygotically, but not maternally. The two Cactus isoforms have a much more limited difference in structure than the isoforms of Dorsal or Dif; roughly speaking, the Cactus isoform arising by intron retention differs only by a truncation of 12 residues at the carboxyl-terminus. This difference is nevertheless of functional consequence, altering stability of the unbound form and, thereby, responsive to Toll signaling [26]. We find it an intriguing possibility that the *dorsal* and *cactus* loci coevolved splice variants that operate in concert with one another in larval tissue.

B isoforms of Dorsal-related NF-κB proteins have been reported for several other insects, including mosquitoes (*Aedes*) and kissing bugs (*Rhodnius*) [33, 34]. We have identified additional orthologs of Dorsal B in the honeybee (*Apis*) and other insects (see S4 Fig). We have not, however, found sequences with B domain similarity among vertebrate NF-κB proteins nor among the Relish family of NF-κB proteins. Given these findings, B isoform function might reflect features of nervous system anatomy and physiology specific to the insects.

The B domain sequences exhibit substantial conservation, but do not have obvious similarity to any known protein motifs. Furthermore, the idea that B isoforms function by heterodimerization with their A isoform counterparts is largely ruled out by our finding that the NMJ and brain appear to express only the B isoform of Dorsal and Dif, respectively. We are therefore left with few clues as to the biochemical and molecular functions of the B domains. However, we note in this context that many genes regulating neuronal system function have been found to undergo alternative splicing in the nervous system [35, 36].

Prior to this study, there were no known mutations specific to the B domain of either *dorsal* or *Dif*. Why? Mutations in *dorsal* and *Dif* have typically been isolated in screens based on phenotypes in dorsoventral patterning or immune function (see, for example, [4, 37, 38]). Evidence suggests that the B isoforms are dispensable for these functions and hence invisible to such screening strategies. Neither Dorsal B nor Dif is detectably expressed in the ovarian tissues that direct embryonic axis formation [7, 16]. Moreover, although both Dorsal isoforms are upregulated upon immune challenge [39], transgenic lines expressing only the A forms of both Dorsal and Dif exhibit wild-type induction of Toll-regulated immune loci, as measured by RNAseq (S. Lindsay, unpublished data).

### Dorsal B expression and function at the NMJ

Cantera and colleagues have described defects in normal neuromuscular function that arise from inactivation of either *dorsal* or *cactus* at the NMJ [40]. Our results indicate that Dorsal B is the only isoform of Dorsal at the NMJ and is therefore required for wild-type NMJ activity.

The Davis group has found that Dorsal regulates the level of the glutamate receptor GluRIIA at the NMJ [15]. Based on their observations regarding particular *dorsal* mutations, we can attribute this effect on GluRIIA to Dorsal B. Specifically, Davis and colleagues found that the GluRIIA level is reduced in all but one *dorsal* mutant background they studied. The exceptional mutation was the *dl^U5^* allele: levels of GluRIIA appeared normal in flies that carried this allele in *trans* to a null mutation. It turns out that *dl^U5^* is a nonsense mutation in the A specific domain of Dorsal (Q488stop). Thus, the one *dorsal* allele that did not have an effect on GluRIIA levels was the one allele that affected Dorsal A, but not Dorsal B.

Although we have studied Dorsal B only at the larval NMJ, its function may not be limited to body wall muscles. We note in this regard evidence from mass spectrometry indicating that Dorsal B is also expressed in the adult heart [41].

### B domain regulation

The amino-terminal half of the Dorsal and Dif B isoforms contains the Rel-homology region, which in A isoforms mediates Cactus binding in the cytoplasm and κB site recognition in the nucleus. Our data indicate that Dorsal B and Dif B lack nuclear localization. How should we interpret this observation? One possibility is that the B isoforms reside and function outside of the nuclear compartment. Consistent with this model, the B isoforms lack the nuclear localization signal (NLS) that is positioned at the carboxyl-terminal end of the RHR of Dorsal A, Dif A, and many other NF-κB proteins. What this model does not explain is the interaction of both Dorsal B and Dif B with Cactus.

An alternative model is that Dorsal B and Dif B undergo regulated nuclear localization *in vivo*, but that the necessary signal is not present in our muscle cell and brain preparations. If, for example, Dorsal B functioned in maintaining or modulating synaptic responsiveness, one might not expect any large-scale movement into nuclei. By this model Cactus might contribute to the regulation of B isoform localization, but it could not be the sole determinant. Given that the B isoforms lack the canonical NLS present in the A isoforms, translocation into the nucleus would need to be governed by other sequence elements [42].

Although Dorsal B and Dif B presumably receive cellular inputs, the nature of that regulation remains ill defined. One likely input is synaptic activity, as demonstrated in studies of Dorsal and Cactus levels at the NMJ [14]. Whether or not Toll signaling influences B isoform activity, the regulation and activity of the Dorsal and Dif B isoforms is clearly noncanonical and worthy of further study.

## Materials and Methods

### Ethics statement

Animal care and procedures were in accordance with the National Institutes of Health recommendations for the humane use of animals. This study was carried out in strict accordance with the Guidelines of the Institutional Animal Care and Use Committee (IACUC) at the University of California, San Diego. The protocol was approved by the IACUC at the University of California, San Diego (Permit Number: S98083). Rabbits were euthanized with ketamine at 35 mg/kg and xylazine at 5 mg/kg.

### *Drosophila* strains

Flies were maintained at 25°C on standard corn meal molasses food. Chromosomal deficiency *Df(2L)J4*, hereafter *DfJ4*, was obtained from William McGinnis. *Df(2L)TW119* and *Df(2L)Exel7068*, which span the *DfJ4* breakpoints, were obtained from the Bloomington Stock Center, as were the *24BGal4* driver and UAS-*Dlg*RNAi strains. UAS-*cactus*RNAi (5848R-3) was obtained from NIG-FLY (Kyoto, Japan). Ethan Bier provided *ms1096*Gal4. Donald J. van Meyel provided UAS-Dorsal A.

### Generation of wild-type and mutant Dif transgenes

To generate isoform-specific transgenes, we used a recombineering approach [43, 44]. The Dorsal and Dif genes lie within a 40 kb region on chromosome 2L of *D*. *melanogaster*. Using inverse PCR and then sequencing, we mapped the precise breakpoints of *DfJ4*, which removes both *dorsal*, *Dif*, and seven additional loci [9]. Starting with BAC clone RP98-7M13, we then used recombineering-mediated gap repair to generate a 43 kb subclone in the attB-P[acman]-ApR plasmid that spanned the 39 kb region deleted in *DfJ4*.

Next, we carried out site-specific mutagenesis of this subclone to eliminate expression of specific Dif isoforms by altering or eliminating splice sites (see S7 Fig). Using ØC31-mediated transgenesis, we introduced these mutant Bac subclones, as well as the wild-type (*J4rescue*), into the *Drosophila* genome at an attP landing site located at 86Fb (BDSC stock #24749). Finally, we generated stable lines with two identical transgene copies in a background null for *dorsal* and *Dif* (*Df(2L)J4*/ *Df(2L)Exel7068*). The net result was a wild-type gene set for *dorsal* and the replacement of both copies of Dif with a wild-type or mutant transgene.

### Generation of UAS-*dorsalA_*RNAi and UAS-*dorsalB_*RNAi strains

Transgenic strains encoding UAS-directed shRNA specific for *dorsalA* or *dorsalB* were made according to the protocol on the TRiP website (www.flyrnai.org/TRiP-HOME.html). The sequences chosen for making short hairpin RNAs were: GCAGCAGUACGACAAUACUGC for *dorsalA* and CAGUGAGAUUGAACAGCAAGU for *dorsalB*. The DNAs encoding the shRNAs were cloned in the pWALIUM20 vector [45] and the resulting constructs underwent ØC31-mediated integration into the *attP* target site at 86F on the polytene map [46, 47]

### Immunohistochemistry and antibodies

To generate B isoform-specific antibodies, we immunized rabbits with bacterially expressed GST fusion proteins containing sequence unique to the B isoform of either Dorsal or Dif (One rabbit per antigen, New Zealand white, 5 lb female).

The *Drosophila* larval NMJ preparation and staining was carried out as previously described [48]. Larval brains were dissected and stained as previously described [49], except that the fixation was done with 4.0% paraformaldehyde in PBS overnight at 4°C. The following primary antibodies were used: mouse α-DorsalA (7A4, 1:5, DSHB) [19], rabbit α-Dorsal (1:2000) [20], rabbit α-DorsalB (1:4000, this work), rabbit α-DifB (1:3000, this work), rabbit α-Cactus (1:2000) [50], mouse α-Dlg (DLG1, 1:5, DSHB), chicken α-GFP (A10262, 1:400, Invitrogen), and Alexa Fluor 647-conjugated goat α-HRP (1:400, Jackson Immuno). The following secondary antibodies were used: Alexa Fluor 488-conjugated donkey α-chicken IgG (1:400, Biotium), Alexa Fluor 488-conjugated goat α-mouse or α-rabbit IgG (1:400, Jackson Immuno), and Cy3-conjugated donkey α-mouse IgG or α-rabbit (1:400, Jackson Immuno). Nuclei were stained with DAPI (1 μg/μl, Roche).

### Confocal microscopy

Confocal images were collected on an Olympus FV1000 confocal microscope with 20x dry, 40x oil and 63x oil objectives. All experiments involved a minimum of three larvae, with examination of four muscles on each side of each larva. Multipanel images were assembled with Adobe Photoshop.

## Supporting Information

S1 Fig Monoclonal α-DorsalA efficiently detects Dorsal A in larval tissue. Wing imaginal discs from + (*ms1096Gal4/+*), and Dorsal A overexpression (*ms1096Gal4/*UAS-Dorsal-A) larvae stained with DAPI and α-DorsalA. Scale bar = 10 μm.
(TIF)

S2 Fig Dorsal B localization is unchanged upon elimination of Dlg expression at the SSR. Body wall muscles from + (*24BGal4/+*) and Dlg knockdown (*24BGal4/*UAS-*Dlg*RNAi) larvae labeled with α-Dlg, α-DorsalB and α-HRP. *Dlg* RNAi using the pan-muscle driver *24BGal4* eliminates Dlg expression from the post-synaptic compartment; residual Dlg staining is pre-synaptic. Images are of muscle 4. Scale bar = 10 μm.
(TIF)

S3 Fig Alternative splicing of the *dorsal*, *Dif*, and *cactus* genes gives rise to intron retention. (TIF)

S4 Fig The B domains of *D*. *melanogaster* Dorsal and Dif are evolutionarily conserved in insects. Alignment (TCoffee) of the B domains from Dorsal and Dif orthologs from the following species: *Drosophila melanogaster*, *Drosophila mojavensis*, *Aedes aegypti*, *Culex quinquefasciatus*, *Apis mellifera*, *Solenopsis invicta*, *Rhodnius prolixus*, *Nasonia vitripennis*, and *Musca domestica*.
(TIFF)

S5 Fig B domain alignment continued. (TIFF)

S6 Fig B domain alignment continued. (TIFF)

S7 Fig Site-directed mutagenesis of J4 rescue constructs. Schematic of mutations introduced into the *Dif* gene of the J4 rescue construct to eliminate splice form A or B.
(TIF)

S1 File ARRIVE guidelines checklist. (PDF)
