# Supplementary figures and images for: Alternative NF-κB Isoforms in the Drosophila Neuromuscular Junction and Brain
Source: PLoS One. 2015 Jul 13;10(7):e0132793. doi: 10.1371/journal.pone.0132793 (PMC4500392; doi:10.1371/journal.pone.0132793)

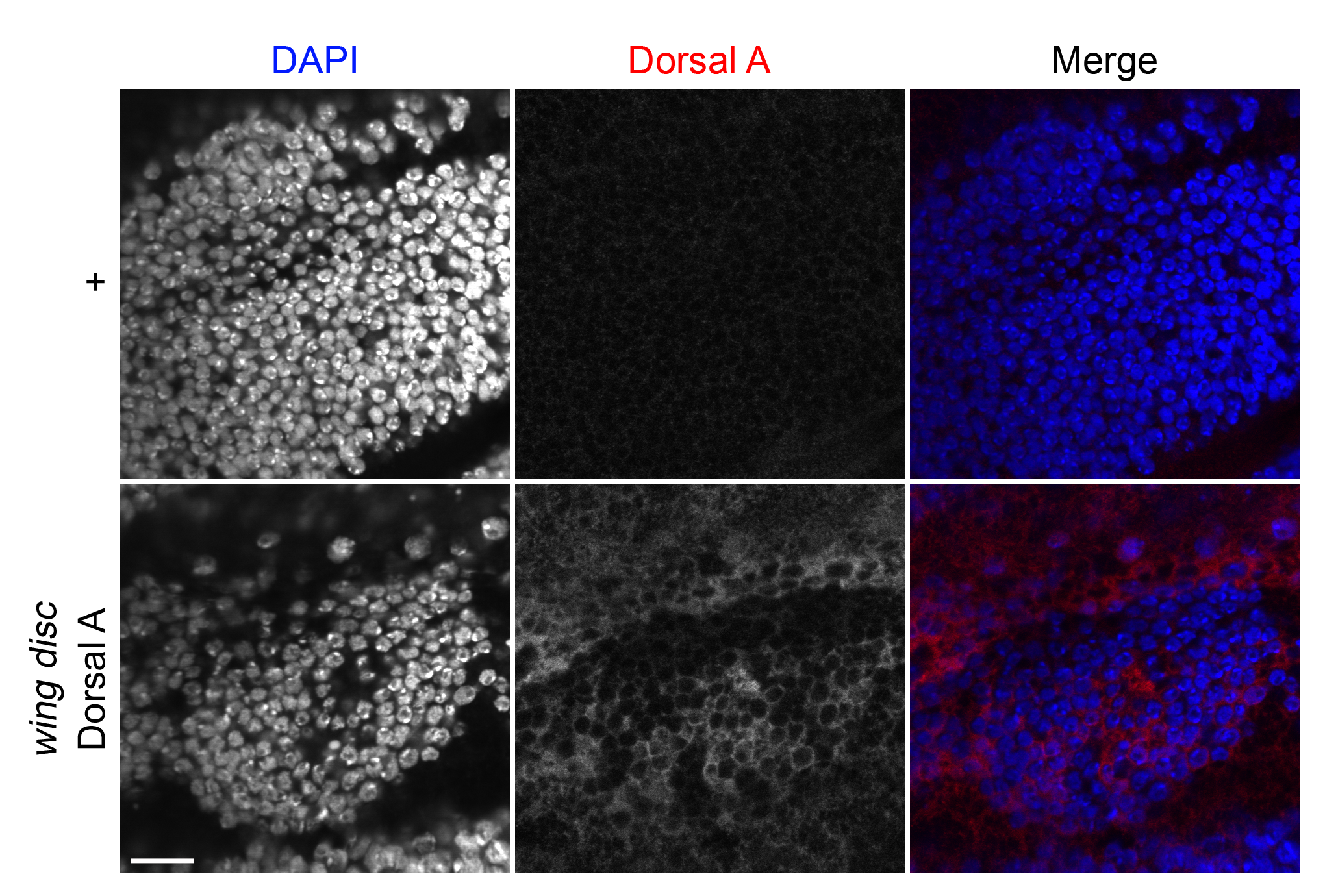

Supplement: S1 Fig — Wing imaginal discs from + (ms1096Gal4/+), and Dorsal A overexpression (ms1096Gal4/UAS-Dorsal-A) larvae stained with DAPI and α-DorsalA. Scale bar = 10 μm. (TIF) [file pone.0132793.s001.tif]

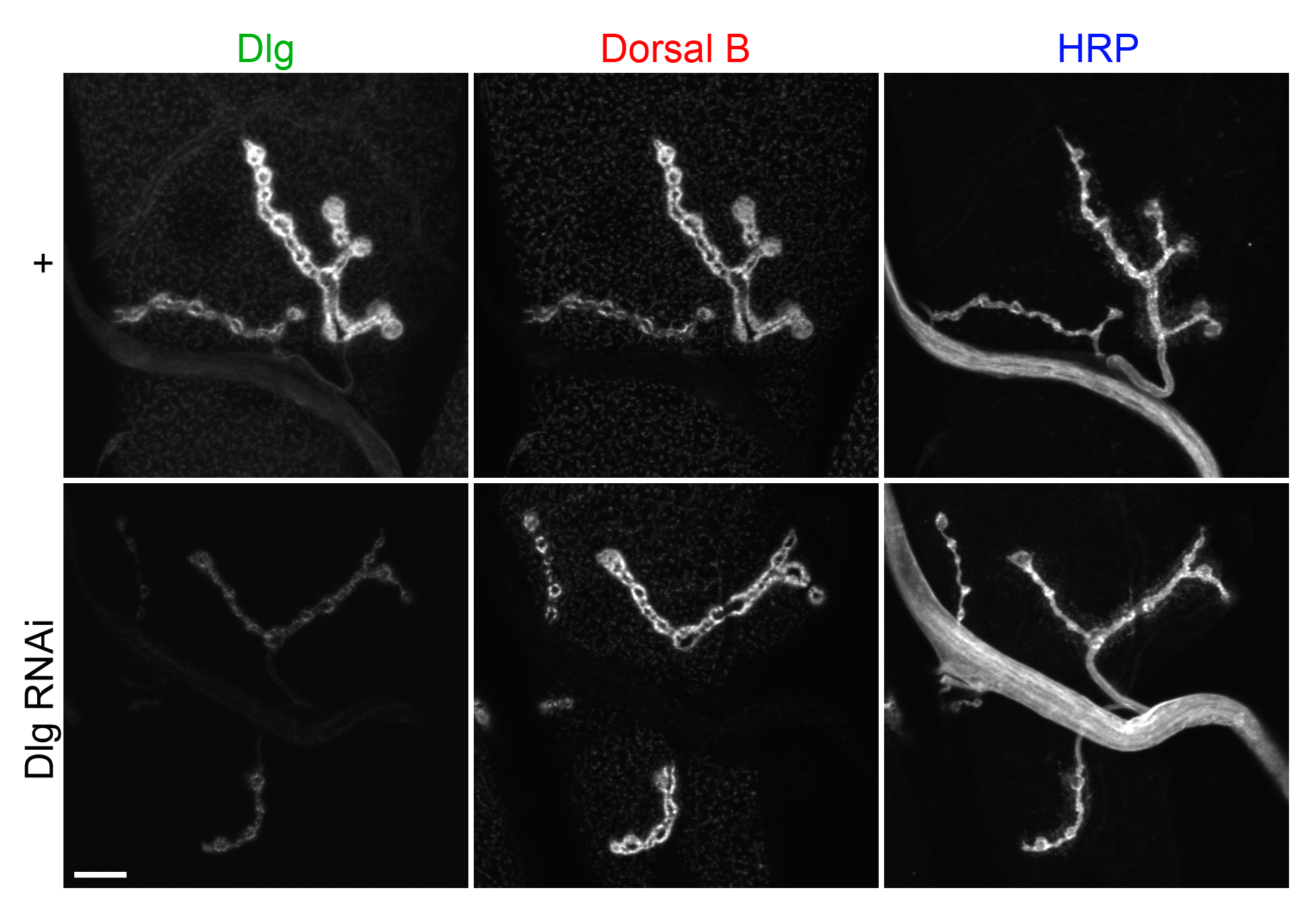

Supplement: S2 Fig — Body wall muscles from + (24BGal4/+) and Dlg knockdown (24BGal4/UAS-DlgRNAi) larvae labeled with α-Dlg, α-DorsalB and α-HRP. Dlg RNAi using the pan-muscle driver 24BGal4 eliminates Dlg expression from the post-synaptic compartment; residual Dlg staining is pre-synaptic. Images are of muscle 4. Scale bar = 10 μm. (TIF) [file pone.0132793.s002.tif]

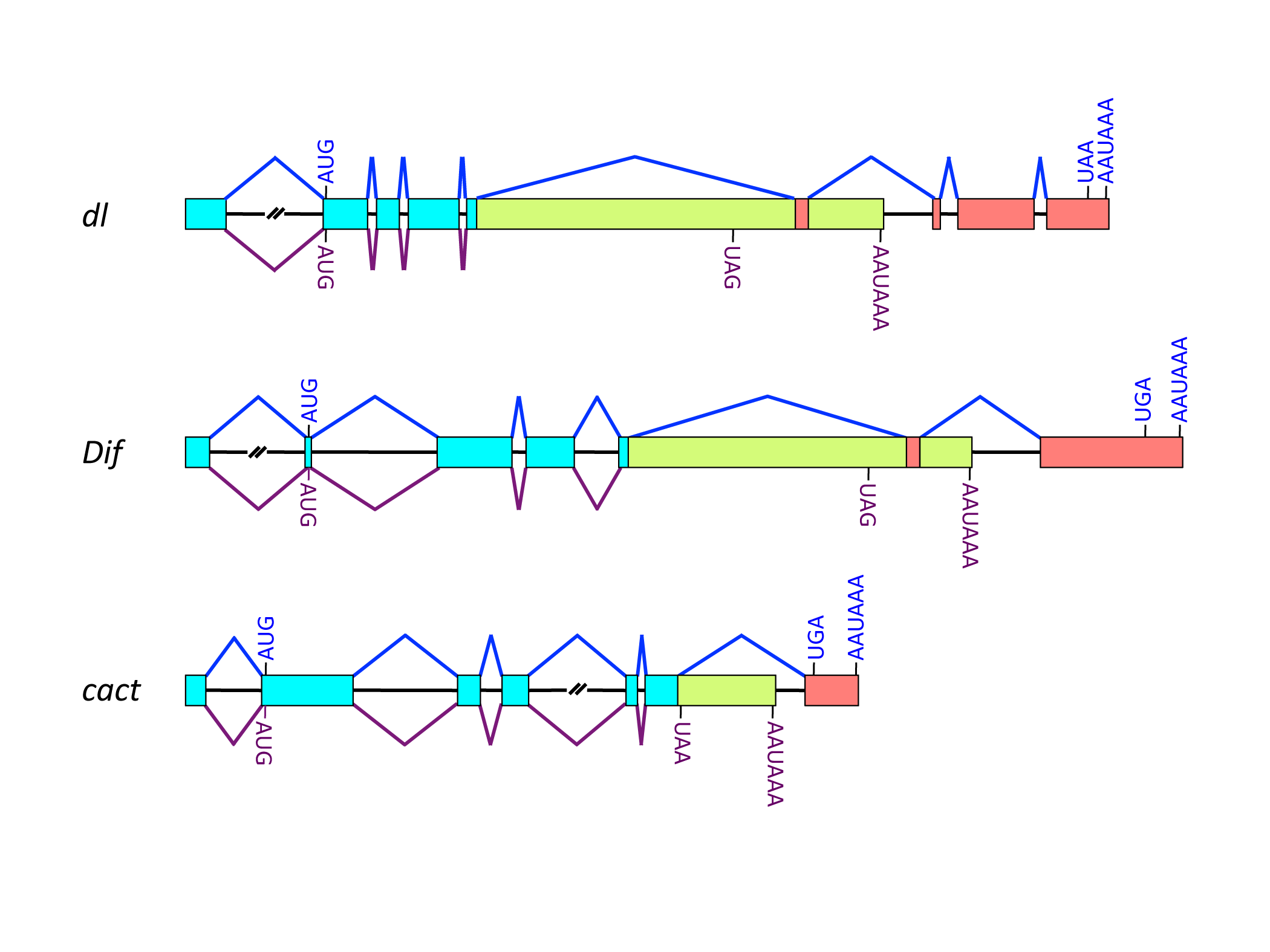

Supplement: S3 Fig — (TIF) [file pone.0132793.s003.tif]

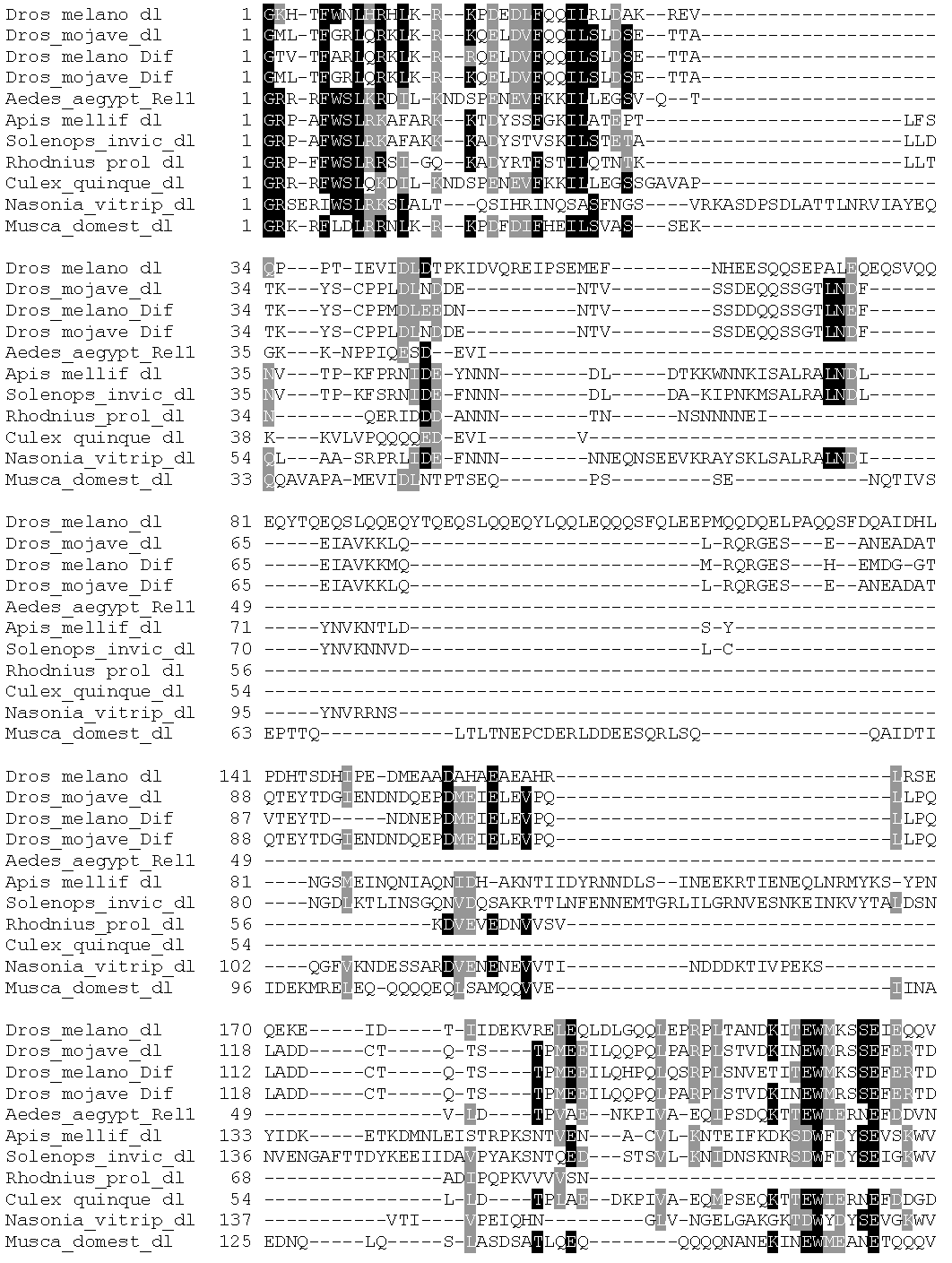

Supplement: S4 Fig — Alignment (TCoffee) of the B domains from Dorsal and Dif orthologs from the following species: Drosophila melanogaster, Drosophila mojavensis, Aedes aegypti, Culex quinquefasciatus, Apis mellifera, Solenopsis invicta, Rhodnius prolixus, Nasonia vitripennis, and Musca domestica. (TIFF) [file pone.0132793.s004.tiff]

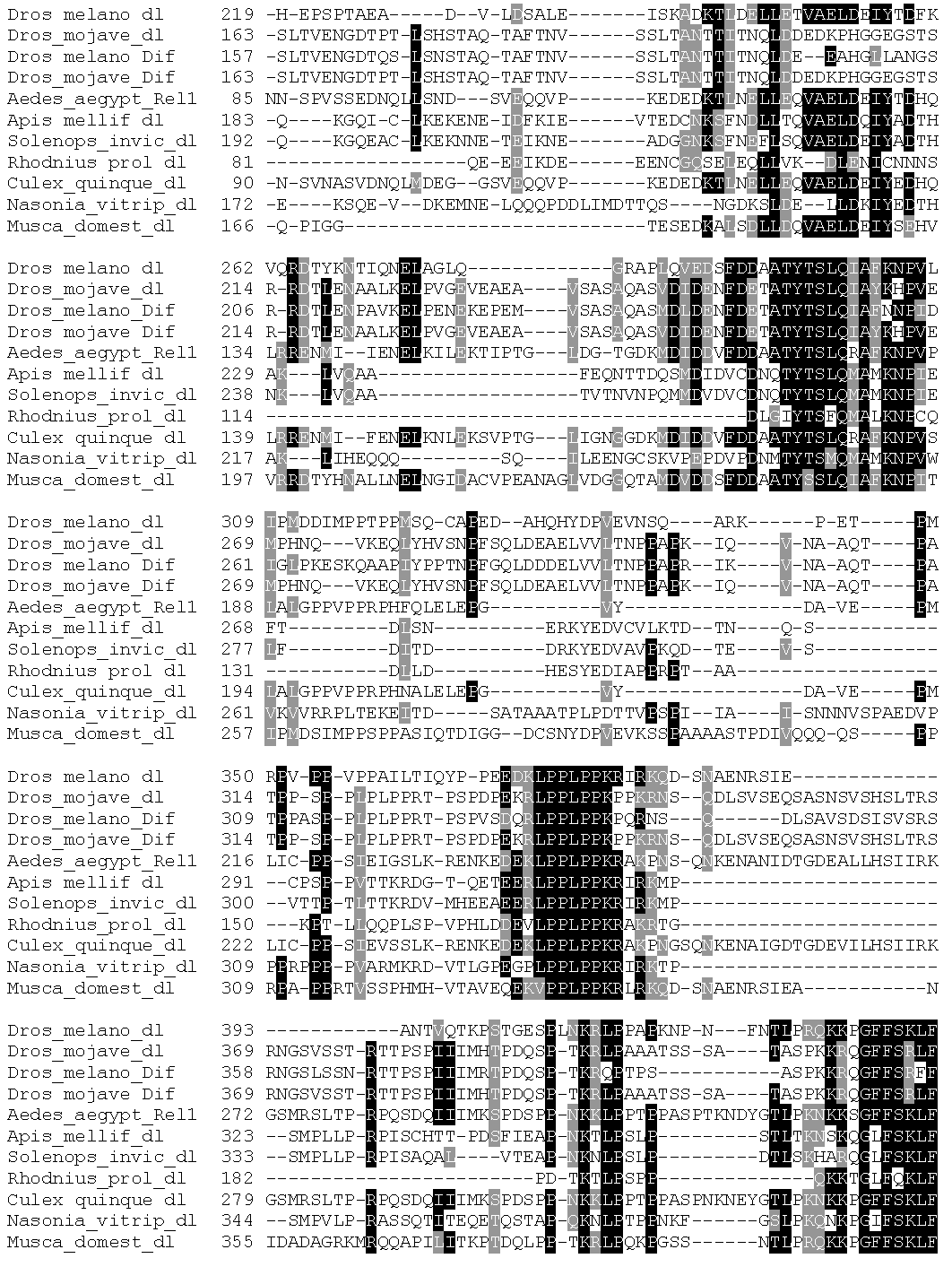

Supplement: S5 Fig — (TIFF) [file pone.0132793.s005.tiff]

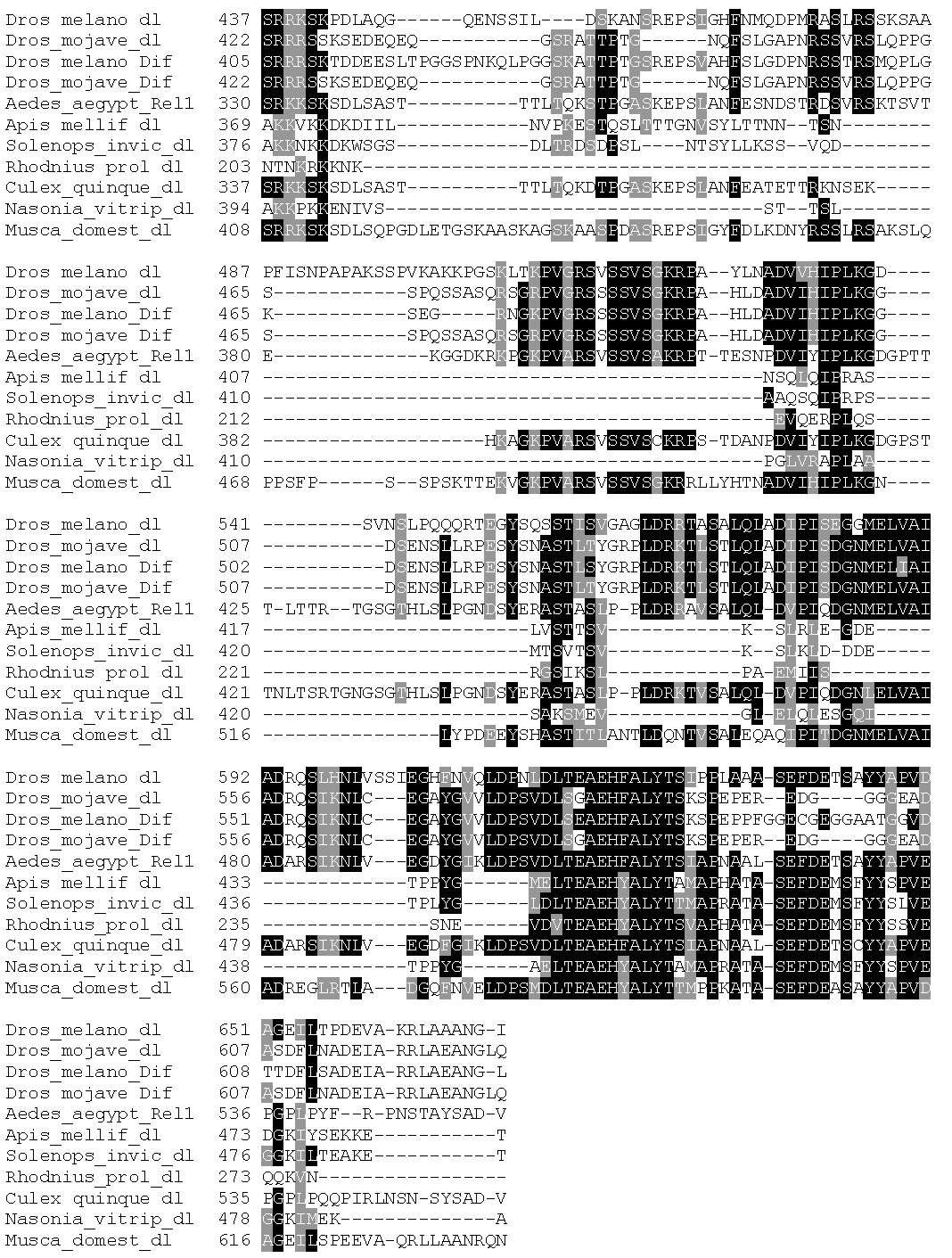

Supplement: S6 Fig — (TIFF) [file pone.0132793.s006.tiff]

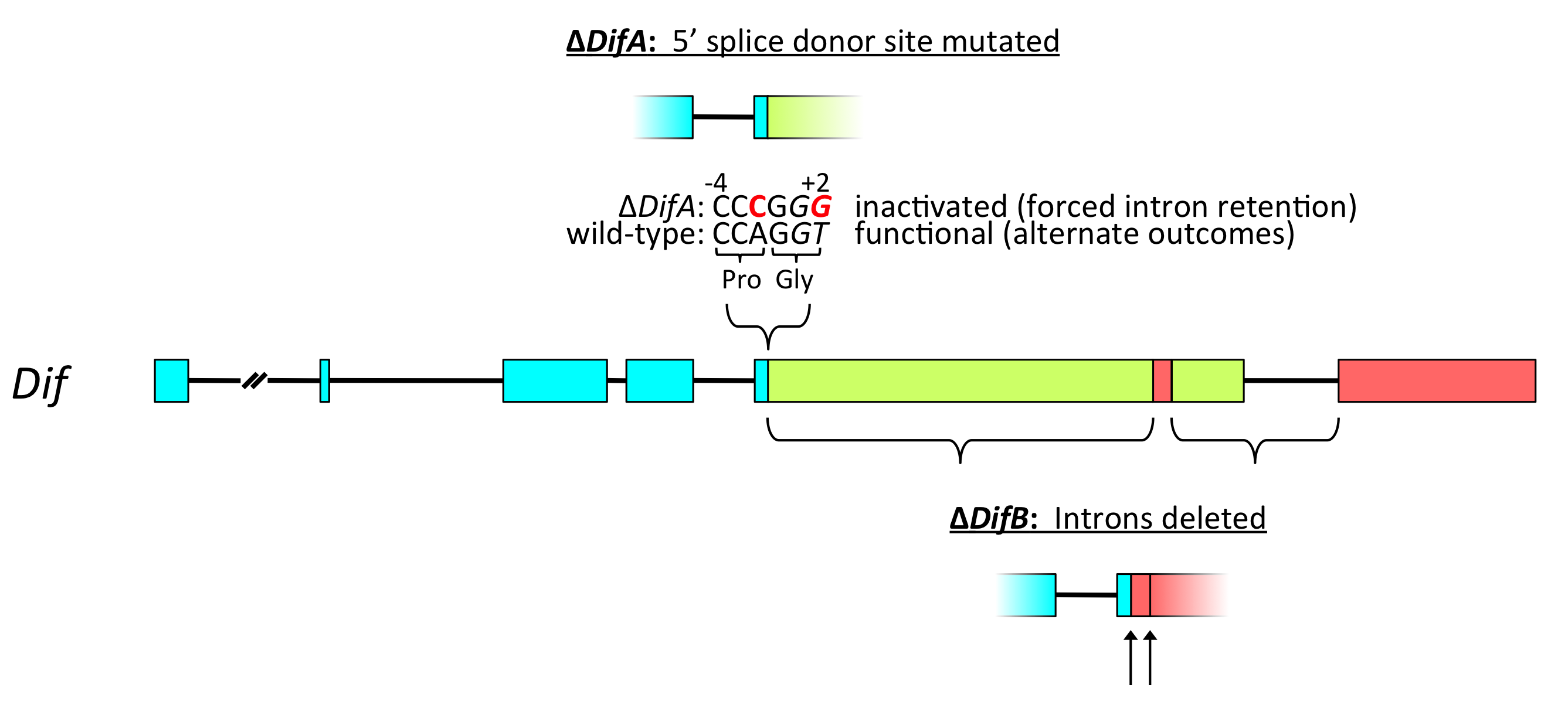

Supplement: S7 Fig — Schematic of mutations introduced into the Dif gene of the J4 rescue construct to eliminate splice form A or B. (TIF) [file pone.0132793.s007.tif]
